# Identification of GCC-box and TCC-box motifs in the promoters of differentially expressed genes in rice (*Oryza sativa* L.): Experimental and computational approaches

**DOI:** 10.1371/journal.pone.0214964

**Published:** 2019-04-26

**Authors:** Gopal Kumar Prajapati, Bharati Pandey, Awdhesh Kumar Mishra, Kwang-Hyun Baek, Dev Mani Pandey

**Affiliations:** 1 Department of Bio-Engineering, Birla Institute of Technology, Mesra, Ranchi, Jharkhand, India; 2 Department of Biotechnology, Yeungnam University, Gyeongsan, Gyeongbuk, Republic of Korea; Clemson University, UNITED STATES

## Abstract

The transcription factor selectively binds with the *cis*-regulatory elements of the promoter and regulates the differential expression of genes. In this study, we aimed to identify and validate the presence of GCC-box and TCC-box motifs in the promoters of upregulated differentially expressed genes (UR-DEGs) and downregulated differentially expressed genes (DR-DEGs) under anoxia using molecular beacon probe (MBP) based real-time PCR. The GCC-box motif was detected in UR-DEGs (*DnaJ* and *60S ribosomal protein L7* genes), whereas, the TCC-box was detected in DR-DEGs (*DnaK* and *CPuORF11* genes). In addition, the mechanism of interaction of AP2/EREBP family transcription factor (LOC_Os03g22170) with GCC-box promoter motif present in *DnaJ* gene (LOC_Os06g09560) and *60S ribosomal protein L7* gene (LOC_Os08g42920); and TCC-box promoter motif of *DnaK* gene (LOC_Os02g48110) and *CPuORF11* gene (LOC_Os02g01240) were explored using molecular dynamics (MD) simulations analysis including binding free energy calculations, principal component analyses, and free energy landscapes. The binding free energy analysis revealed that AP2/EREBP model residues such as Arg68, Arg72, Arg83, Lys87, and Arg90 were commonly involved in the formation of hydrogen bonds with GCC and TCC-box promoter motifs, suggesting that these residues are critical for strong interaction. The movement of the entire protein bound to DNA was restricted, confirming the stability of the complex. This study provides comprehensive binding information and a more detailed view of the dynamic interaction between proteins and DNA.

## Introduction

Standing crops face various stresses during their life cycle, which result in a drastic reduction in yield [1]. Although some crops withstand environmental stresses by developing new features, others are unable to develop adaptive mechanisms and consequently die. Importantly, rice has a lower tolerance and higher susceptibility to abiotic stresses than other crops [2,3]. In plants, low oxygen stress stimulates composite metabolic pathways and genetic programs, including the differential expression of several genes [4]. Gene expression studies have revealed the upregulation of genes encoding transcription factors, as well as signal transduction components [5]. For example, a wide range of differentially expressed genes (DEGs) have been studied using microarray analyses [6], and the expression pattern of 23 proteins and their respective mRNAs has been analyzed in anoxic rice coleoptile [7].

In *Arabidopsis*, ETHYLENE RESPONSE FACTOR (AtERF) positively or negatively responds by binding specifically to AGCCGCC sequence known as GCC-box or to its substituted form TCC-box and modulate the gene expression in response to biotic and abiotic factors [8,9]. GCC-box is also found in the promoters of many pathogen-responsive genes such as PDF1.2 and PR regulates specific defense phenomena [9,10]. APETALA2/ethylene response factor (AP2/ERF) plant transcription factor genes regulate developmental processes and are involved in the responses to various biotic and abiotic stresses [11]. Furthermore, the AP2/ERF family of transcriptional regulators with the Sub1A-1-mediated response plays important role in submergence tolerance [12]. The differential expression of 163 *AP2/EREBP*(APETALA 2/ethylene responsive element-binding protein)genes in rice under abiotic stress conditions has been studied [13]. Kumar et al. [14] reported the presence of a consensus promoter motif with a conserved GCC-box (GCCGCC) in the upregulated differentially expressed genes (UR-DEGs) using publicly available microarray data for anoxic rice coleoptile [6]. Likewise, another study reported the presence of a TCC-box (TCCTCC) in the promoters of downregulated DEGs (DR-DEGs) in anoxic rice coleoptiles [14,15]. Techniques for the detection of specific nucleic acid sequence, probe-based like Molecular Beacon Probe (MBP), TaqMan, Minor groove binding (MGB) is being used by various researchers [16–18]. However, MBP is more sensitive and precision based detection over conventional PCR without post-reaction analysis [19]. More importantly, MBP probe differentiates with single nucleotide differences which increase high specificity over TaqMan [20]. Also, unlike TaqMan probes, MBP are designed in such a way so that they remain intact during the amplification reaction and capable to rebind with target in every cycle for signal measurement [21].

Promoter motifs/*cis*-regulatory elements are involved in the regulation of differentially expressed genes, and regulates cellular mechanisms in response to abiotic and biotic stresses. Thus, the identification of differentially expressed genes and the mechanisms underlying differential expression is of great interest. The presence of consensus motifs, such as a GCC-box, in UR-DEGs and TCC-box in DR-DEGs needs to be validated using a sequence-based technique by designing motif sequence-specific MBPs and performing MBP based real-time PCR analyses. Real-time PCR data can be analyzed using the Ct value, which is the number of cycles required for the fluorescent signal to cross a threshold [16–18]. GCC-box and TCC-box of DEGs has important role in the transcriptional regulation of genes during various stress [8,9,11,15]. Therefore, in this study, we aimed to use MBP based real-time PCR assays to accurately detect GCC-boxes in UR-DEGs such as *DnaJ* (LOC_Os06g09560) and *60S ribosomal protein L7* (LOC_Os08g42920), and TCC-boxes in DR-DEGs such as *DnaK* (LOC_Os02g48110) and *CPuORF11* (LOC_Os02g01240). In the recent scenario, Molecular Dynamics (MD) simulation has proven to be powerful atomistic simulation algorithms for predicting interaction strength between two macromolecules [22]. MD simulations have been extensively applied in elucidating residues responsible for transcription factor and DNA motif. WRKY transcription factor-DNA complex interaction using 10 ns MD simulations in *A*. *thaliana* have been studied [23]. In a similar study, important structural features stabilizing DOF zinc finger-DNA complexes using *in silico* approaches have also been identified [24]. In addition, Pandey et al. [25] have studied the AP2-DNA interaction in barley and found that residues in the beta-strand were crucial for stabilizing the AP2-DNA complex. Therefore, in the present study, we examined the key interactions occurring between AP2/EREBP family transcription factor(LOC_Os03g22170) and GCC and TCC-box DNA motifs using molecular and essential dynamics based binding mechanics analysis.

## Material and methods

### Selection of DEGs and MBP design

Microarray data of DEGs in anoxic rice coleoptiles [6] and a dataset of Kumar et al [14] were used to shortlist UR-DEGs and DR-DEGs for analysis in this study. The UR-DEGs and DR-DEGs were ranked based on their expression score ≥2 fold (≥2X) and ≤-2 fold ≤ -2X), respectively. The promoter sequences -499 to +100 bp of the selected UR-DEGs and DR-DEGs were retrieved from the Eukaryotic Promoter Database as described previously [14]. The retrieved promoter regions were analyzed using the MEME (Multiple Em for Motif Elicitation) web server (http://meme-suite.org/tools/meme). Furthermore, the consensus promoter motif of UR-DEGs and DR-DEGs were used to design MBPs using Beacon Designer 7 (BD7, PREMIER Biosoft, USA). Custom made MBPs and primers were procured from Gene Link, (New York, USA). The methodology used for rice genomic DNA isolation and the validation of the consensus promoter motif is described in our previous work. It is well established that the AP2/EREBP transcription factor (TF) DNA-binding domain (DBD) binds to GCC-box [12,13,15,26]. The AP2/EREBP TF model from rice was generated using SWISS-MODEL web server [27] and the structure quality was assessed using PROCHECK [28] based on the Ramachandran plot. A three dimensional (3D) structural model of the DNA motif was generated using 3D-DART (3DNA-Driven DNA Analysis and Rebuilding Tool) [29]. Five 3D DNA models of GCC- (CGCCGCCGCCG) and TCC-box motifs (CTCCTCCTCCTCCTC) were generated with a bend angle of 0–40°. 3D-DART enables the generation of DNA models based on customized local and global conformations, such as the bend angle range and bend angle orientation range.

### High ambiguity driven protein-DNA docking

For the protein-DNA interaction study, DNA models of gene promoter motifs (GCC- and TCC-box) were docked onto the specific site of the AP2/EREBP TF using the HADDOCK (High Ambiguity Driven protein-protein Docking) web server (version 2.2) [15,26,30]. Residues 68, 69, 71, 73, 75, 77, 82, 83, 90, 92, 94, 95, 108, 109, and 110 were considered as active site residues for the protein, and 1-50 base pair (bp) nucleotides from both DNA stands were selected as active residues for the DNA motif. Passive residues were spontaneously defined around active residues. In reference to active and passive residues, Ambiguous Interaction Restraints (AIR) was generated. Here, illustration and visualization of the final docked complex were completed using UCSF Chimera [31].

### Molecular dynamics simulations for the protein and docked complexes

To study the dynamics and recognition mechanism between AP2/EREBP TF and DNA motifs, the generated complexes were subjected to MD simulations using the GROMACS 5.0 software package [32,33]. OPLS-AA/L all-atom force field and AMBER99SB-ILDN force field were applied to AP2/EREBP TF and protein-DNA complexes simulations, respectively [34]. Furthermore, systems were solvated in a minimal cubic water box using the Simple Point Charge (SPC) water model [35]. Solvated systems carry a charge; therefore, ions were added to neutralize the entire system by substituting water molecules with ions. The systems were energy minimized (50000 cycles of steepest descent) to remove steric clashes and inappropriate geometry. The minimized systems were equilibrated (the solvent and ions around the protein needed to be equilibrated) into NVT (constant Number of particles, Volume, and Temperature) and NPT (constant Number of particles, Pressure, and Temperature) phases for 1000 ps [25, 36–38]. The well-equilibrated systems were then subjected to a production run at 300 K and 100000-pascal pressure for 50,000 ps. The analyses of the 50 ns MD trajectories were carried out using GROMACS built-in tools. The various interactions involved in the pre- and post-MD of protein-DNA simulated complexes were deduced using Nucplot [39].

The stability of the complex was calculated by measuring the RMSD (root mean square deviation) of the protein backbone atoms’ positions with respect to the start or reference structure using the following equation:

$$\text{RMSD(t)}={\left[\frac{1}{M}\sum_{i=1}^{N}m_{i}|r_{i}(t)-r_{i}^{ref}{|}^{2}\right]}^{\frac{1}{2}}$$ (1)

where M=Σ_i_ m_i_ and r_i_(t) is the position of atom i at time t after least square fitting the structure to the reference structure. The RMSF (root mean square fluctuations) was calculated using the following equation:

$$RMSF_{i=}{\left[\frac{1}{T}\overset{}{\underset{}{\sum_{T_{j=1}}^{T}{\left|r_{i\left(t_{j}\right)-r_{i}^{ref}}\right|}^{2}}}\right]}^{\frac{1}{2}}$$ (2)

where T is the time over which one wants to average and r_i_^ref^ is the reference position of particle i.

### Binding free energy and free energy decomposition analysis

The package g_mmpbsa calculates the binding energy of bimolecular associations such as protein-protein, protein-ligand, and protein-DNA associations using the Molecular Mechanics Poisson-Boltzmann Surface Area (MM-PBSA) protocol [40]. It provides the different components of energy terms such as polar solvation, non-polar solvation, and electrostatic energy. The MmPbSaDecomp.py python script was used to determine the residue-wise contribution to the total binding energy, which provides information about important residues contributing to the molecular association.

### Principal component analysis (PCA) and free energy analysis

Principal component analysis (PCA) is widely used to gain insights into the adequate structural and dynamics of the protein and complex trajectories [41]. PCA is a multivariate statistical analysis used to extract covariant motions on a number of different lengths and time scales from a protein structure. The covariance matrix of the atomic fluctuations was calculated using the gmx-covar module of gromacs software and calculated using the following equation:

$$C_{ij}=\left\lfloor M_{II}^{\frac{1}{2}}\left(x_{i}-\left(x_{i}\right)\right)M_{jj}^{\frac{1}{2}}\left(x_{j}-\left(x_{j}\right)\right)\right\rfloor$$

In which, C implies 3n x 3n symmetric matrix, n is a number of residues and M is a diagonal matrix [42].

Diagonalization of this matrix yields a set of eigenvectors and eigenvalues that describe collective modes of fluctuations of the protein. The eigenvectors corresponding to the largest eigenvalues are called “principal components”, as they represent the largest-amplitude collective motions. The eigenvectors were analyzed using the gmx-anaeig gromacs built-in command. The gmx-sham tool was used to generate the input for free energy landscapes using the axes of a principal component analysis.

## Results and discussion

### GCC-box and TCC-box detection and validation

**Fig 1:**
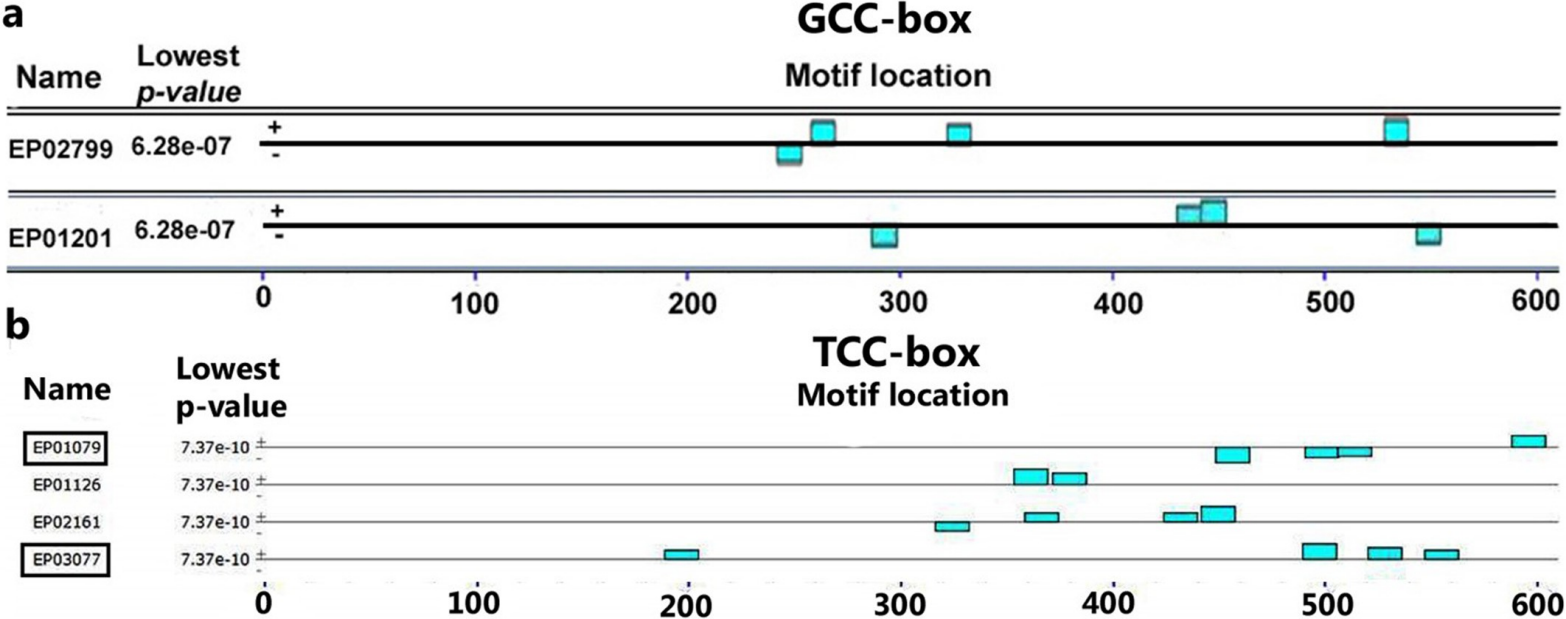
The position of promoter motifs in various genes. (a) GCC-boxes in the promoters of *DnaJ* (EP01201 or LOC_Os06g09560) and *60S ribosomal protein L7* genes (EP02799 or LOC_ Os08g42920); (b) TCC-box in *DnaK* (EP03077 or LOC_Os02g48110) and *CPuORF11* (EP01079 or LOC_Os02g01240) genes.

Under anoxia UR-DEGs with expression by equal or higher than two-fold (≥2X) and expression by equal or lower than -2 fold (≤ -2X) for DR-DEGs were selected from the microarray results [6] and the aforementioned datasets [14]. The selected UR-DEGs and DR-DEGs were analyzed using MEME (v 4.5.0) to identify consensus promoter motifs (GCC-box and TCC-box). We identified the presence of GCC-box and TCC-box motifs in the promoter region of UR-DEGs (*DnaJ* and *60S ribosomal protein L7*) and DR-DEGs (*DnaK* and *CPuORF11*), respectively. The GCC-box motif was acknowledged in the *DnaJ* (EP01201) and *60S ribosomal protein L7* (EP02799) genes with the lowest p-value of 6.28e^-07^ indicates the most significant match score of the given motifs (Fig 1A). Similarly, TCC-box motifs were identified in *DnaK* (EP03077) and *CPuORF11*(EP01079) genes with the lowest p-value 7.37e^-10^ (Fig 1B).

Gene expression studies revealed the upregulation of genes encoding transcription factors under hypoxic response in *Arabidopsis* [5]. However, the regulation of gene expression occurs through the core promoter motif sequence [43]. Promoter motifs contain specific nucleotide sequences that are responsible for gene regulation and function under different biotic and abiotic conditions. Hence, the identification and validation of these regulatory elements are essential. Expression analysis of the *60S ribosomal protein L7* has been used as an internal control for gene expression studies in *Coffea arabica* under different experimental conditions [44]. DnaJ, which contains a J domain of 70 amino acid consensus sequence, is a co-chaperone of Hsp70 (DnaK) and facilitates Hsp70’s ATPase activity, substrate delivery, and specific cellular localization [45]. In *Arabidopsis* and rice, J proteins have been implicated in the protection against environmental stresses [46]. DnaK family proteins also include heat shock proteins that are involved in protecting plants against abiotic stresses [47]. *CPuORF11*, which has an ORF found in the 5' UTR of a mature mRNA, mediate translational regulation in response to sucrose concentration, amino acid production, starvation and polyamine concentration. However, it’s mechanism of action is not clearly raised in *Arabidopsis* and Rice [48–50]. A sequence of GCC-box and TCC-box repeats was used to design a molecular beacon probe. Forward and reverse primers were designed (Table 1) using the parameters and compatibility in Beacon Designer 7. The MBPs designed for UR-DEGs and DR-DEGs were 5’-[6-FAM] CGCGATCGCCGCCGCCGGATCGCG [BHQ-1]-3’, and 5’-[6-FAM] CGCGATCCTCCTCCTCCTCCTCGATCGCG [BHQ-1]-3’, respectively. The MBPs included the reporter dye 6-FAM (6-Carboxyfluorescein) at the 5′ end and the quencher BHQ1 (Black Hole Quencher-1) at the 3’ end [15,26]. In the present study, two UR-DEGs (*DnaJ* and *60S ribosomal protein L7*) and two DR-DEGs (*DnaK* and *CPuORF11*) were validated by experimental and computational studies. The presence of GCC- and TCC-boxes in selected genes was verified by real-time PCR assays. We have taken promoter region belongs to TSS (transcription start site) of the selected gene considering promoter position from -499 to +100 i.e., 600 nt and the same region has been used for motif detection by MBP. In *DnaJ* gene promoter is in upstream position i.e., -62 to -52 and in *60S ribosomal protein L7* gene promoter, GCC box position is in downstream i.e., from + 30 to + 40 (Table 2). Similarly, in *DnaK* gene promoter, TCC box position is in upstream -18 to -4 and in *CPuORF11* genes promoter TCC box position is in upstream -58 to -44 (Table 2). Amplification of GCC-box sequences was confirmed by MBP, with average Ct values of 34.21 and 31.65 for *DnaJ* and *60S ribosomal protein L7*, respectively (Table 2). Similarly, TCC-box containing genes were amplified by MBP, with average Ct values of 27.79 and 28.5 for *DnaK* and *CPuORF11*, respectively (Table 2).

**Table 1:** List of primers designed for UR-DEGs and DR-DEGs.

DEGs		Forward	Reverse	Amplicon size
***DnaJ* (EP01201)**		5′-CGTGAGTGAGTCTTCCGTGTCTTC3′	5′-GCCACCGAGCACCTGTCC-3′	137
***60S ribosomal protein L7*** **(EP02799)**		5′-GCCATAATAAGACGGTGAGA-3′	5′-CCGCTATCTCTACGCAAG-3′	112
***DnaK* (EP03077)**		5′-TTCAGCAGCAACGCACAA-3′	5′-GGAGAGAGCAGCGAA GGA-3′	173
***CPuORF11* (EP01079)**		5′-GAGTGATCCGTTATATCTGTT5′	5′-CTCTCCTTCCTTCCTTC T-3′	200

**Table 2:** Promoter motif position, strand position and Ct values of UR-DEGs (*DnaJ*, *60S ribosomal protein L7*) and DR-DEGs (*DnaK*, *CPuORF11*) amplified using MBPs specific to the GCC-box and TCC-box motifs.

DEGs	MBP	Motif position	Strand position	Replicates	Ct value	Average Ct value
***DnaJ***	GCC box	-62 to -52449	+strand	R1	34.07	34.21
				R2	34.34	
***60S ribosomal protein L7***	GCC box	+30 to + 40	+strand	R1	32.17	31.65
				R2	31.12	
***DnaK***	TCC box	-18 to -4	+strand	R1	28.04	27.79
				R2	27.54	
***CPuORF11***	TCC box	-58 to -44	- strand	R1	28.28	28.5
				R2	28.71	

In rice, the Submergence1 (Sub1) locus encodes three ethylene-responsive factor (ERF), transcriptional regulators. It has been described that a large member of the ERF family interacts specifically with AGCCGCC through their conserved domain [51]. Direct interaction of GCC-boxes and non-GCC-boxes with Tomato transcription factor Pti4 (an ERF) revealed the involvement of ERFs in gene regulation and expression [52]. The binding of maltose binding protein (AtERF) to the GCC sequence (AGCCGCC) in *Arabidopsis* was hampered when both G residues within the GCC-box were replaced by T (ATCCTCC) [8, 53]. Several reports based on the gene ontology classification and differential expression of *DnaJ*, *60S ribosomal protein L7*, *DnaK* and *CPuORF11* genes in diverse species suggest that these genes are involved in cellular, biological, and molecular functions in the plant. In our previous work, MBP based real-time PCR analysis indicated that UR-DEGs and DR-DEGs under anoxic conditions that contained a GCC-box and TCC-box in their promoter region bound AP2/EREBP TF in rice[15]. Hence, validation of the *in silico* findings of GCC-box and TCC-box promoter motifs in the UR-DEGs (*DnaJ* and *60S ribosomal protein L7*) and DR-DEGs (*DnaK* and *CPuORF11*) in *O*. *sativa* is essential.

### Protein and DNA motif modeling

**Fig 2:**
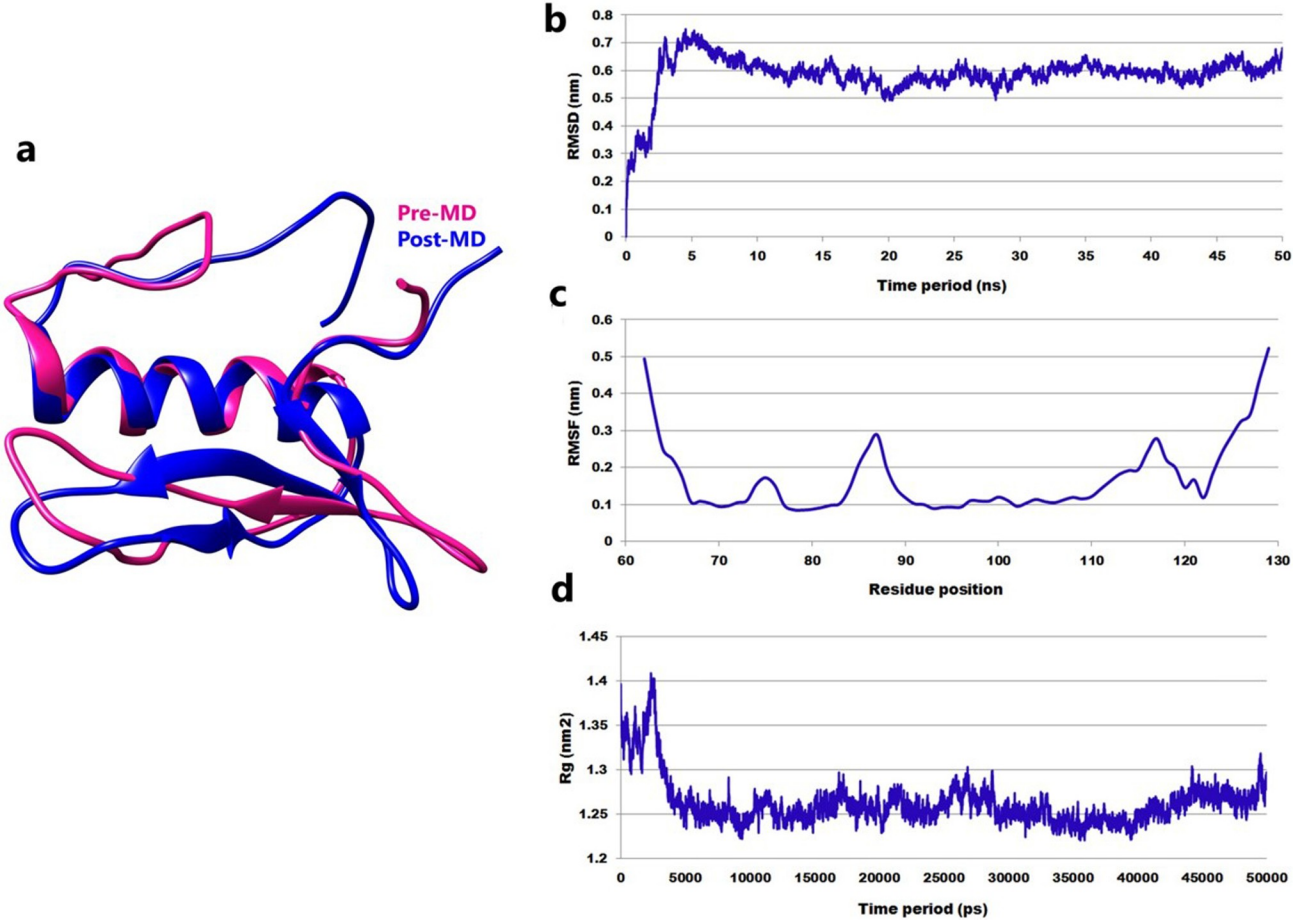
Three-dimensional structure of the rice AP2/EREBP TF and MD simulation. (a) Superimposition of pre- and post-MD simulation AP2/EREBP TF; (b) RMSD analysis; (c) RMSF analysis; (d) radius of gyration for MD simulations with a 50 ns time period.

BLASTP was performed for AP2/EREBP TF sequences (LOC_Os03g22170) against the PDB database. Blast hits showed a 71% sequence identity with an E value of 2e^-21^ to the recently solved crystal structure of AtERF96 containing a GCC-box (resolution: 1.76Å) from *Arabidopsis thaliana* (PDB ID: 5wx9; chain A), which was selected as a template for the construction of the AP2/EREBP TF model (Fig 2A).

Analysis of the stereochemical quality of individual residues in the protein was carried out using Ramachandran Plot. In the generated model, the percentage of residues in the most favored regions and additional allowed regions was 89.7% and 6.9%, respectively. According to the plot, 3.4% of the residues were located in the disallowed region. Analysis of the secondary structure of the AP2/EREBP TF revealed that it consists of one β-sheet, three β-strands, one α-helix, five β-turns, and one gamma (γ) turn (S1A Fig).

### Analysis of molecular dynamics (MD) simulations of the AP2/EREBP TF

Structural refinement was carried out using molecular dynamics (MD) simulations with solvents and ions. Superimposition of pre-and post-MD simulated AP2/EREBP TF revealed a backbone RMSD deviation of 1.17Å (Fig 2A). The AP2/EREBP TF attains equilibrium after 10 ns and sustains the stability until the end of the simulation time period with an average RMSD of 0.59 nm (Fig 2B). RMSF showed a peak for individual residue, and two regions of the protein showed the highest fluctuation; 83-90 and 110-120 residues, whereas the rest of the structure remained stable with an average RMSF value of approximately 0.17 nm (Fig 2C). The radius of gyration of the protein backbone atoms was 1.26 nm, which contributed to the compactness of the protein. The representative structure was extracted from the stable time frame and used for the protein-DNA docking analysis. The simulated structure was analyzed using Ramachandran Plot, which revealed that residues found in the additional allowed regions had increased to 15.5% whereas, residues found in the disallowed region reduced to 1.7%, suggesting that the MD simulations increased the stability of the protein structure [54]. No difference in the secondary structure elements was observed in the pre- and post MD simulated AP2/EREBP TF structures (S1B Fig).

### Protein-DNA interaction and stability analysis

**Fig 3:**
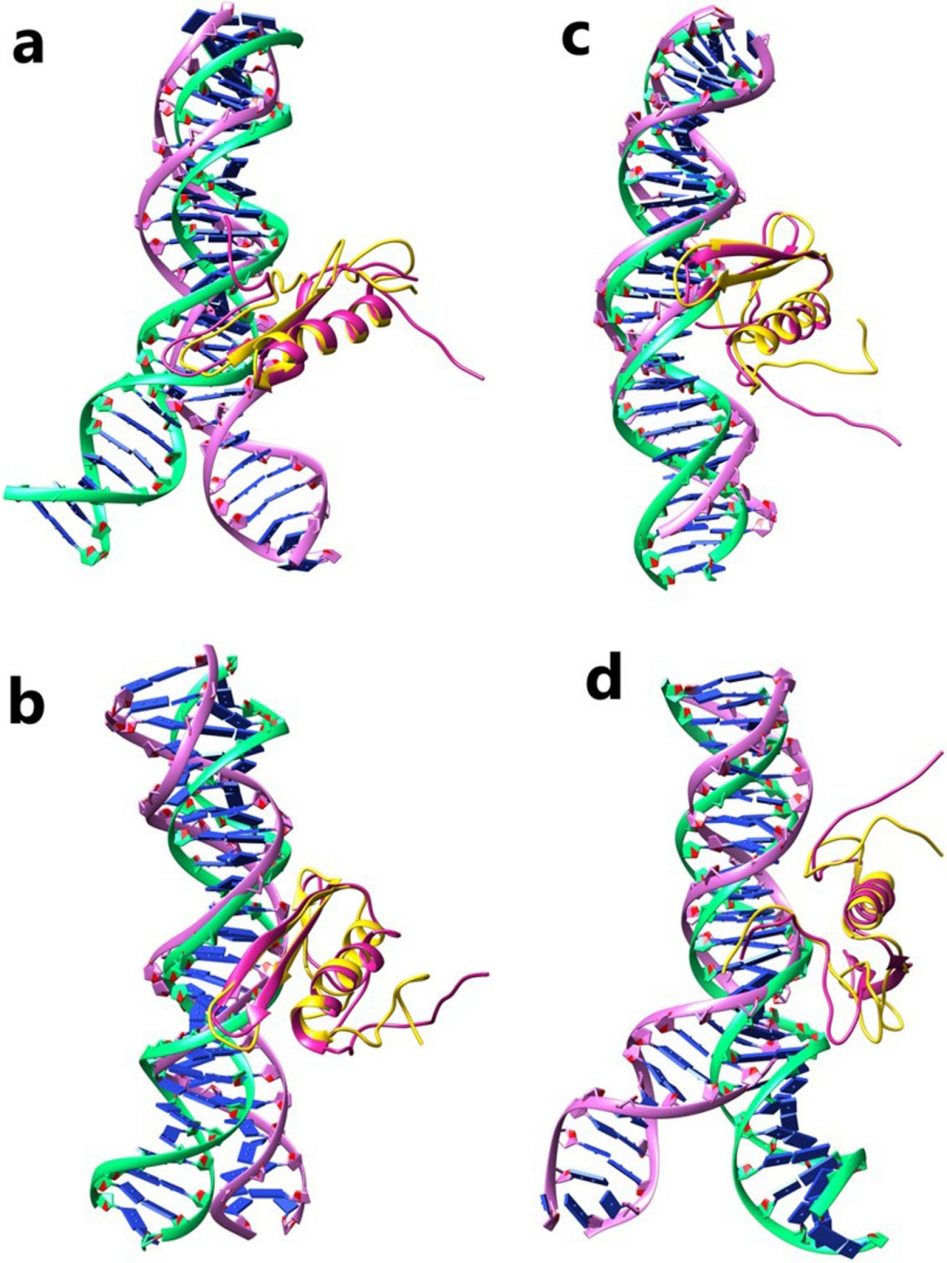
Superimposition of pre- and post-MD simulation complexes. Interactions of the pre-MD and post-MD simulated complexes for (a) IHSAPDTM-BS; (b) IRPAPDTM-BS; (c) IDNAPDTM-BS; and (d) IOFAPBTM-BS. DNA is represented in green (pre-MD) and purple (post-MD), and protein is represented in pink (pre-MD) and gold (post-MD).

To predict which amino acids interact with DNA, the representative structure of the AP2/EREBP TF was docked with a GCC-box and TCC-box using HADDOCK. Protein-GCC-box complexes were named as IHSAPDTM-BS, IRPAPDTM-BS and protein-TCC-box complexes as IDNAPDTM-BS and IOFAPBTM-BS. Both GCC-box and TCC-box motif DNA models were generated with 0° to 40° DNA bend angles (S2A and S2B Fig) and docked individually with the AP2/EREBP TF (S3 and S4). Cluster 1 had a maximum cluster size of 98 with the highest HADDOCK score of -134.2 ± 2.3 and -142.2 ± 3.3 for both IHSAPDTM-BS and IRPAPDTM-BS, respectively (Table 3). The IHSAPDTM-BS complex was stabilized by the formation of five hydrogen bonds (H-bonds) (Arg68, Arg73, Lys77, Lys87, and Thr95) and six hydrophobic interactions (Table 4 and Fig 3A). Similarly, four bonds (Arg64, Arg73, Lys77, and Arg83) and an extensive network of seven hydrophobic interactions reinforced the IRPAPDTM-BS complex stability (Table 4 and Fig 3B). It was evident from the HADDOCK results that DNA bends at 40° in both IHSAPDTM-BS and IRPAPDTM-BS complexes (GCC-box) had a strong affinity for the AP2/EREBP TF.

**Table 3:** Characteristics of HADDOCK interaction analysis of the AP2/EREBP TF with GCC and TCC-box motifs.

	Interaction	HADDOCK score	Cluster size	RMSD	Van der Waals energy	Electrostatic energy	Desolvation energy	Restraints violation energy	Buried Surface Area	Z-Score
**GCC-BOX**	IHSAPDTM-BS	-134.2 ± 2.3	98	1.1 ± 0.8	-67.1 ± 6.0	-442.0 ± 37.5	20.2 ± 1.9	10.7 ± 12.83	1648.8 ± 99.0	-2
	IRPAPDTM-BS	-142.2 ± 3.3	98	1.9 ± 1.5	-67.9 ± 3.5	-495.2 ± 32.3	24.4 ± 1.9	4.1 ± 1.73	1723.4 ± 72.9	-2
**TCC-box**	IDNAPDTM-BS	-144.2 ± 2.8	33	1.6 ± 1.5	-65.5 ± 6.2	-631.8 ± 36.9	41.1 ± 5.5	65.4 ± 29.74	2000.4 ± 113.3	-1.6
	IOFAPBTM-BS	-147.5 ± 7.3	39	2.8 ± 1.6	-55.4 ± 6.2	-694.5 ± 49.5	43.0 ± 4.7	38.1 ± 21.90	1769.1 ± 124.5	-2

**Keys**: I-Interaction; AP-AP2/EREBP (LOC_Os03g22170) TF; HS-Heat Shock protein DnaJ gene promoter DNA segment (LOC_Os06g09560); RP-60S ribosomal protein L7 gene promoter DNA segment (LOC_Os08g42920); DN-DnaK gene promoter DNA segment (LOC_Os02g48110); OF-CPuORF11-conserved peptide uORF transcript gene promoter DNA segment (LOC_Os02g01240); (A/B/C/D)/T- 10–-40º bend angle; M-Model; BS- binding site.

**Table 4:** List of residues involved in the formation of hydrogen bonds and hydrophobic interactions in AP2/EREBP TF -DNA complexes.

Protein-DNA complex		Residues involved in hydrogen bonding	Residues involved in hydrophobic interactions
**IHSAPDTM-BS**	Pre-MD	Arg68, Arg73, Lys77, Lys87, Thr95	Arg71, Arg72, Trp75, Arg83, Arg90, Trp92
	Post-MD	Arg68, Arg73, Lys87, Arg90, Thr95	Arg73, Trp92
**IRPAPDTM-BS**	Pre-MD	Arg64, Arg73, Lys77, Arg83	Gly69, Arg71, Pro74, Trp75, Lys87, Arg90, Trp92
	Post-MD	Arg71, Arg72, Arg73, Trp75, Arg83, Lys87, Thr95	Arg90, Trp92
**IOFAPBTM-BS**	Pre-MD	Arg64, Arg68, Gly69, Arg71, Arg72, Arg83, Arg90, Lys117, Lys119	Glu62, Arg63, Arg73
	Post-MD	Glu62, Arg63, Arg64, Arg72, Arg83, Lys87, Arg90	Gly69, Arg73
**IDNAPDTM-BS**	Pre-MD	Glu62, Arg63, Arg64, Arg68, Arg71, Arg73, Thr106, Lys119	Leu66, Gly69, Pro74, Lys117, Pro123
	Post-MD	Glu62, Arg63, Arg64, Thr65, Arg68, Arg71, Thr106, Lys117	Gly69, Arg83, Arg114, Lys119, Pro123

The highest HADDOCK score for IDNAPDTM-BS and IOFAPBTM-BS (TCC-box) complexes were found to be -144.2 ± 2.8 and -147.5 ± 7.3, respectively (Table 3). The number of hydrogen bonds and hydrophobic interactions in IOFAPBTM-BS and IDNAPDTM-BS complexes were nine (Arg64, Arg68, Gly69, Arg71, Arg72, Arg83, Arg90, Lys117, and Lys119) and three, and eight (Glu62, Arg63, Arg64, Arg68, Arg71, Arg73, Thr106, and Lys119) and five, respectively (Table 4 and Fig 3C and 3D). The cluster size and Z-score for the selected clusters were 33 and -1.6 for IDNAPDTM-BS, and 39 and -2.0 for IOFAPBTM-BS, respectively. DNA bends at 40° and 20° in IDNAPDTM-BS and IOFAPBTM-BS complexes had strong binding affinities. The HADDOCK results were selected for further MD simulations. Therefore, the conformation adopted by DNA play a very significant role in specific interaction between AP2/EREBP TF and DNA [55].

### Conformational and interaction analysis of the docked complexes after MD simulations

**Fig 4:**
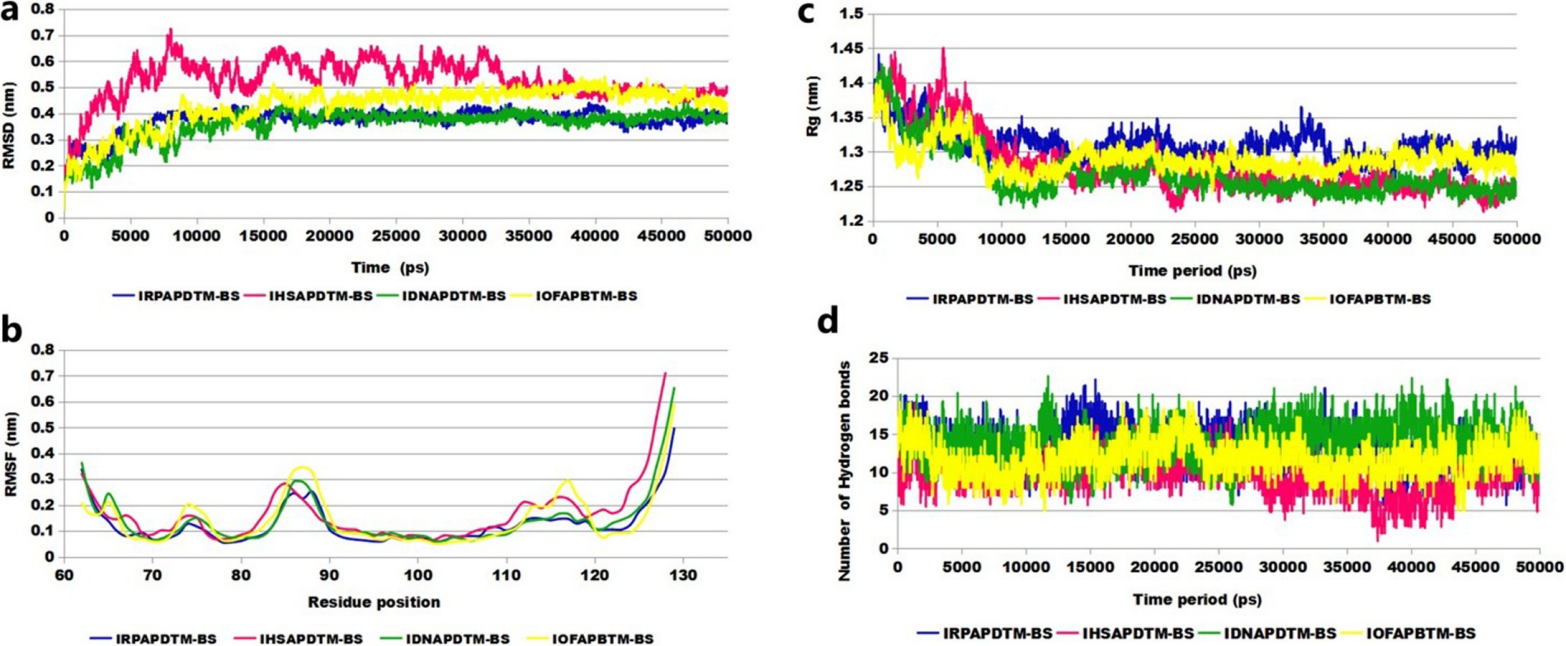
MD simulation trajectory analysis of the AP2/EREBP TF bound to GCC-box and TCC-box motifs. (a) RMSD analysis; (b) RMSF analysis; (c) radius of gyration; and (d) number of hydrogen bonds during the 50 ns MD simulation time period.

To examine the dynamics and to gain specific interaction information, the protein-DNA complexes were subjected to 50 ns MD simulations. IHSAPDTM-BS and IRPAPDTM-BS attained a final conformation with a backbone RMSD of approximately 0.53 nm and 0.37 nm, respectively (Fig 4A). In addition, IDNAPDTM-BS and IOFAPBTM-BS showed an average deviation from the initial structure of 0.36 nm and 0.43 nm, respectively (Fig 4A). RMSD value for the backbone atoms less than 1.0nm suggested stability of the complex structures [56]. Furthermore, the structural deviations of the DNA-bound complexes were analysed at regular time intervals across the simulation trajectory (S1 Table).

The RMSF value of key residues stabilizing the IHSAPDTM-BS (Arg68, Arg73, Lys87, Arg90, and Thr95) and IRPAPDTM-BS (Arg71, Arg72, Arg73, Trp75, Arg83, Lys87, and Thr95) complexes varied from 0.08 to 0.25 nm, respectively (Fig 4B). The RMSF value for the interacting residues in IOFAPBTM-BS (Glu62, Arg63, Arg64, Arg72, Arg83, Lys87, and Arg90) and IDNAPDTM-BS (Glu62, Arg63, Arg64, Thr65, Arg68, Arg71, Thr106, and Lys117) ranged from 0.07 to 0.34 nm, respectively (Fig 4B). Moreover, the radius of gyration and the hydrogen bond analysis for all four complexes indicated the compactness and stability of the complexes (Fig 4C and 4D). MD analysis results indicated that all four complexes underwent minor conformational changes during the simulation time period. The representative docked complexes were extracted from the stable time frame for the identification of key interacting residues.

A comparative interaction analysis was carried out for all protein-DNA complexes. The total number of hydrogen bonds remained unchanged in pre- and post-MD simulated IHSAPDTM-BS and increased from four to seven in IRPAPDTM-BS complexes (Table 4). However, in the IOFAPBTM-BS, the number of hydrogen bonds decreased from nine to seven but remained constant for the IDNAPDTM-BS complex (Table 4). In subsequent MD simulations, the number of hydrophobic interactions reduced drastically in all complexes (IHSAPDTM-BS, IRPAPDTM-BS, and IOFAPBTM-BS) except IDNAPDTM-BS (Table 4). Most of the interacting residues in the pre-simulated complex were conserved in the post-simulated structures, suggesting that they play a crucial role in the formation of AP2/EREBP TF -DNA complex.

### Conformation analysis of the complexes

To study the conformational variation during MD simulations, we extracted snapshots of each complex at 10 ns intervals (0ns, 10 ns, 20 ns, 30 ns, 40 ns, and 50 ns) and analyzed these for the IHSAPDTM-BS, IRPAPDTM-BS, IDNAPDTM-BS, and IOFAPBTM-BS complexes (S3 and S4 Figs). The analysis revealed that the amino acid residues involved in the formation of hydrogen bonds (H-bond) with the DNA remained stable and consistent after 10 ns (S2 Table). Thus, the overall MD simulation trajectory analysis along with the comparative interaction analysis at regular time intervals, indicated that there was a fairly stable interaction between the AP2/EREBP TF and DNA motif through H-bonding and hydrophobic interactions [57].

### Binding free energy analysis

Calculation of protein-DNA binding free energy is a very vast field of research and computational techniques. MM-PBSA method uses the last 5 ns (45–50 ns) of MD simulation trajectories to calculate the binding free energy components, including van der Waal energy, electrostatic energy, polar and non-polar energies and their contribution towards protein-DNA complex stability. The total binding free energy for the IHSAPDTM-BS, IRPAPDTM-BS, IDNAPDTM-BS, and IOFAPBTM-BS complexes were computed to be -27488.958±372.317 kJ/mol, -31225.294±467.742 kJ/mol, -28791.293±438.664 kJ/mol, and -31168.009±438.691 kJ/mol, respectively, high negative binding free energy values suggested strong binding affinity between the AP2/EREBP TF and DNA motifs (Table 5).

**Table 5:** Binding free energy calculation for the AP2/EREBP TF complex with GCC-box and TCC-box motifs.

Protein-DNA complex	Van der Waals (kJ/mol) ΔGvdW	Electrostatic (kJ/mol) ΔGcoul	Polar contribution (kJ/mol) ΔGpolar	Non-polar contribution (kJ/mol) ΔGnonpolar	Binding energy (kJ/mol) ΔG
IHSAPDTM-BS	-234.378 ±18.944	-28944.093 ±412.867	1727.964 ±115.540	-38.452 ± 2.856	-27488.958 ±372.317
IRPAPDTM-BS	-305.842 ±22.860	-33044.70 ±519.260	2165.584 ±159.310	-40.328 ± 2.464	-31225.294 ±467.742
IDNAPDTM-BS	-333.626 ±24.718	-30568.986 ±465.522	2157.740 ±161.546	-46.421 ± 2.424	-28791.293 ±438.664
IOFAPBTM-BS	-213.779 ± 23.833	-33277.075 ±568.813	2354.455 ±207.294	-31.610± 2.974	-31168.009 ±438.691

**Fig 5:**
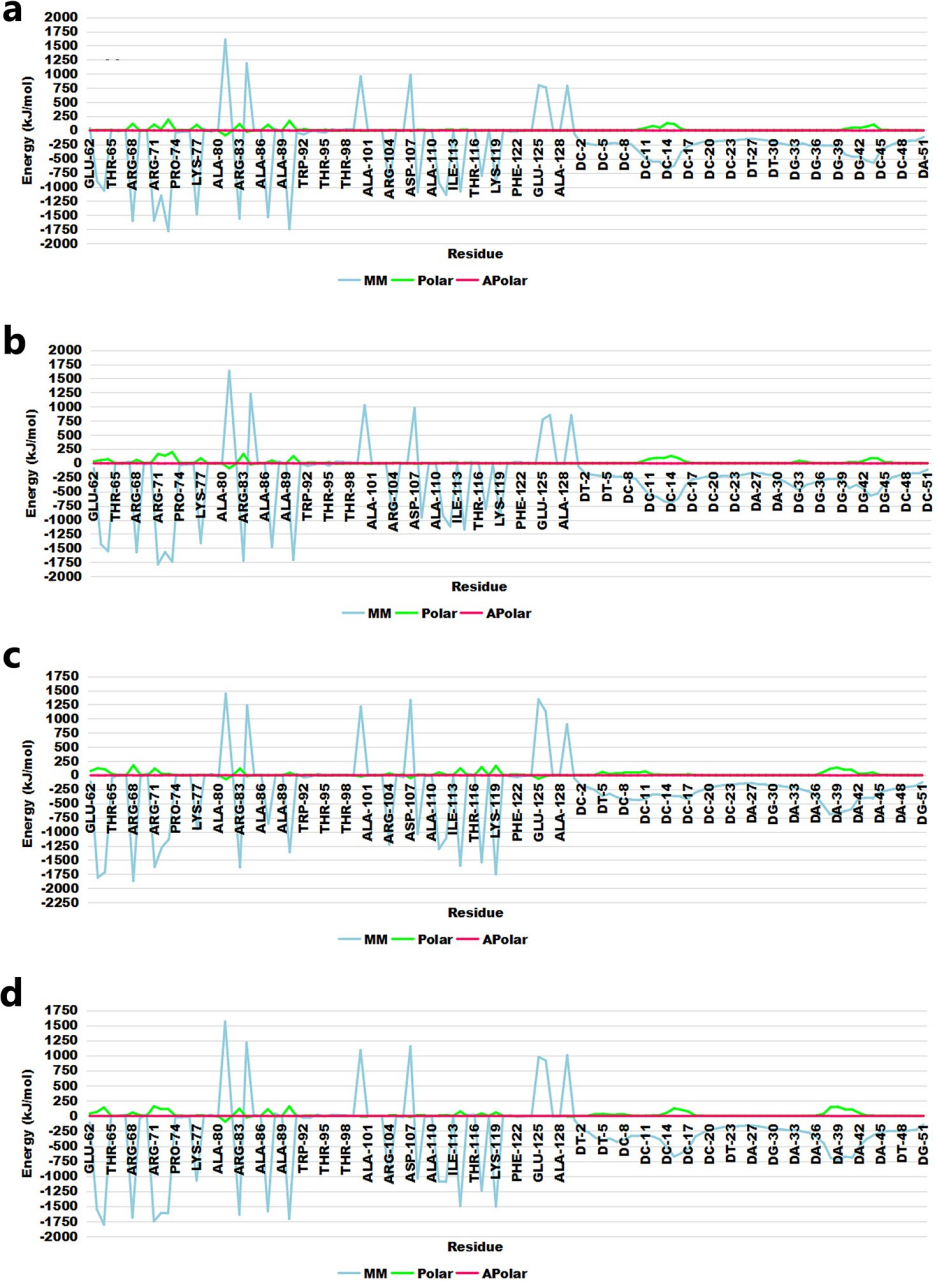
Decomposition of binding free energy per amino acid residue. (a) IHSAPDTM-BS; (b) IRPAPDTM-BS; (c) IDNAPDTM-BS; and (d) IOFAPBTM-BS complexes.

The effect of each residue to the binding energy was computed and showed that the contribution of most of the common interacting residues (Arg68, Arg72, Arg83, Lys87, and Arg90) was observed to be very similar in DNA-bound complexes, suggesting a significant role for these residues in complex stabilization(Fig 5A–5D). Highest contributions were made by electrostatic energy, followed by polar energy. The high binding energy profile was in agreement with the interaction profile of each DNA-bound complex.

### Analysis of conformational fluctuation in AP2/EREBP TF and DNA- bound complexes

**Fig 6:**
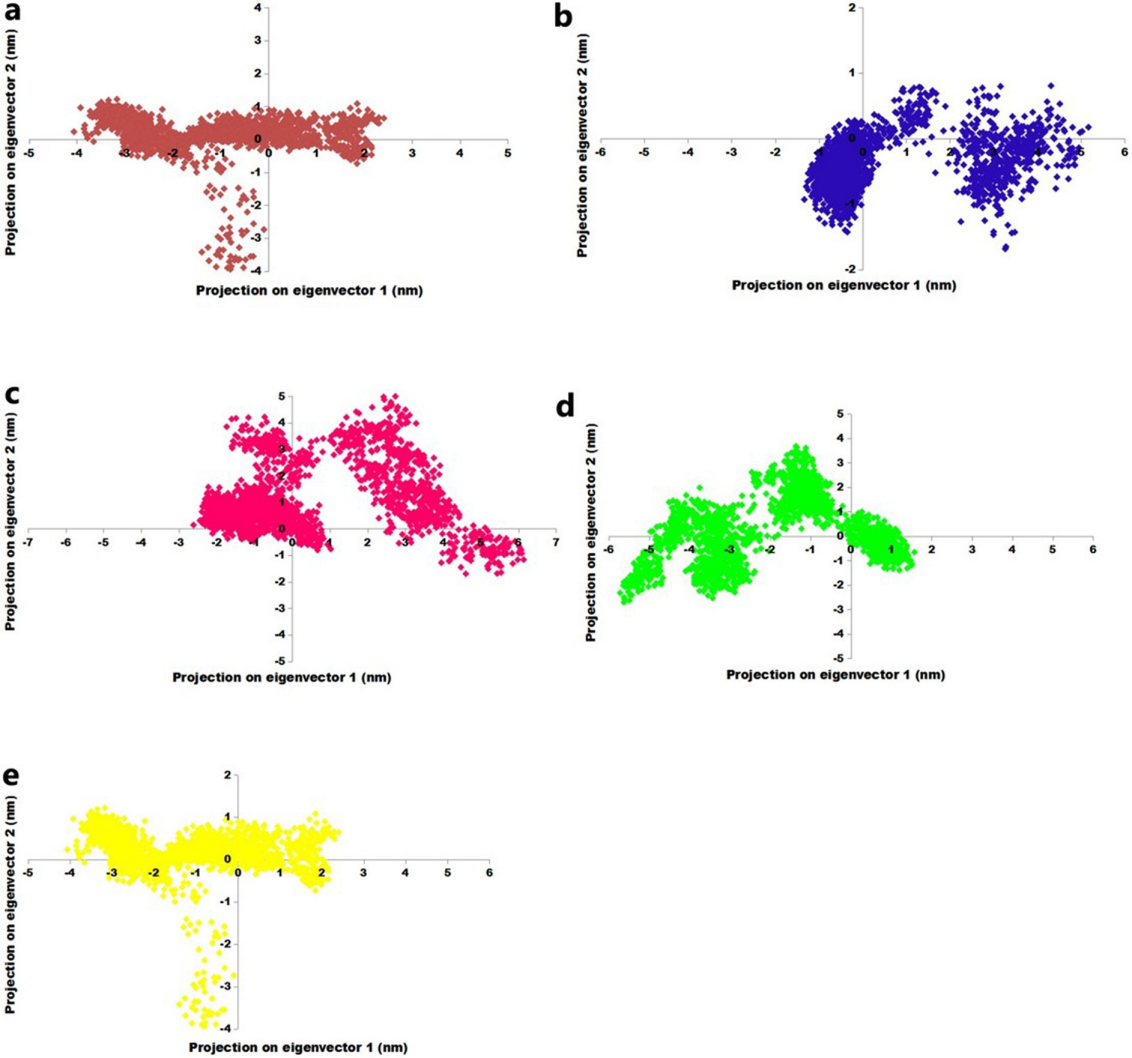
Principal component analysis for the unbound and bound structures. (a) AP2/EREBP TF; (b) IHSAPDTM-BS; (c) IRPAPDTM-BS; (d) IDNAPDTM-BS; and (e) IOFAPBTM-BS.

**Fig 7:**
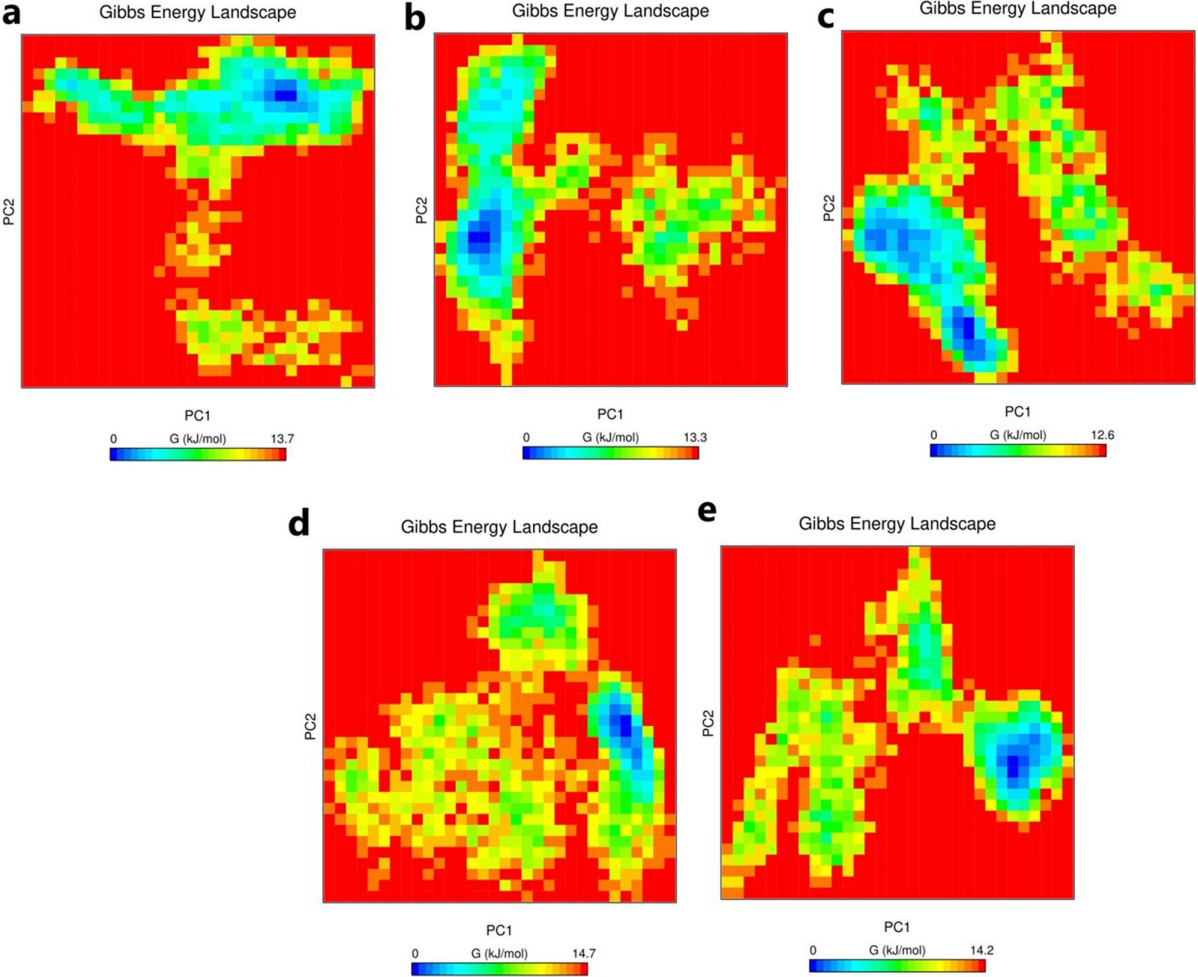
Gibbs free energy landscape for the unbound and bound structures. (a) AP2/EREBP TF (b) IHSAPDTM-BS; (c) IRPAPDTM-BS; (d) IOFAPBTM-BS; and (e) IDNAPDTM-BS.

The development of multivariate methods, such as PCA, promises to enrich the analysis of MD data and to reveal quantitative insights into the relationships between structure, dynamics, and function. Covariance provides information about the cooperativity of motion and can be positive or negative, however, the trace is the sum of the leading diagonal, therefore, and the trace is the sum of the individual variances [58]. The trace value for the AP2/EREBP TF, IHSAPDTM-BS, IRPAPDTM-BS, IDNAPDTM-BS, and IOFAPBTM-BS was 7.6 nm^2^, 8.2 nm^2^, 4.5 nm^2^, 6.3 nm^2^, and 6.2 nm^2^, respectively; the small trace values corresponded to positive covariance and confirmed the decrease in flexibility in the collective motion of the protein, thus revealing a higher stability (Fig 6). The covariance matrix was used to generate the eigenvector and its corresponding eigenvalues for the AP2/EREBP TF and DNA-bound complexes (S5 Fig). The Gibbs free energy (∆G) value ranged from 12.6 to 14.7 kJ/mol for DNA-bound complexes. The overall results indicated the stability of the AP2/EREBP TF and its DNA-bound complexes (Fig 7).

## Conclusion

We successfully designed MBP and specific primers for UR-DEGs (*DnaJ* and *60S ribosomal protein L7*) and DR-DEGs (*DnaK* and *CPuORF11*) and validated the presence of GCC-box and TCC-box promoter motifs. The molecular dynamics study of the protein-DNA complexes revealed a high binding affinity of the AP2/EREBP TF for GCC- and TCC-box motifs in selected genes. The GCC-box amino acid residues Arg68, Arg71, Arg72, Arg73, Trp75, Arg83, Lys87, Arg90 and Thr95, and the TCC-box amino acid residues Glu62, Arg63, Arg64, Thr65, Arg68, Arg71, Arg72, Arg83, Lys87, Arg90, Thr106, and Lys117 directly interacted with DNA. Consequently, these residues play an important role in the stabilization of the complex and the regulation of the differential expression of these genes in rice. Therefore, our results shed light on the underlying mechanism of GCC-box and TCC-box recognition by proteins.

## Supporting information

S1 Fig Secondary structure analysis of the AP2/EREBP TF before and after MD simulations. (TIF)

S2 Fig DNA motif bend angle of 0°, 10°, 20°, 30°, and 40° for an (a) GCC-box; and (b) TCC-box.
(TIF)

S3 Fig Extracted snapshots of (a) IHSAPDTM-BS and (b) IRPAPDTM-BS complexes at regular intervals during the 50 ns simulation time period.
(TIF)

S4 Fig Extracted snapshots of (a) IDNAPDTM-BS and (b) IOFAPBTM-BS complexes at regular intervals during the 50 ns simulation time period.
(TIF)

S5 Fig Covariance analysis of the (a) AP2/EREBP TF; (b) IHSAPDTM-BS; (c) IRPAPDTM-BS; (d) IOFAPBTM-BS; and (e) IDNAPDTM-BS.
(TIF)

S1 Table RMSD of AP2/EREBP TF -DNA complexes at different time intervals. (DOCX)

S2 Table List of residues involved in the formation of hydrogen bonds in AP2/EREBP TF-DNA complexes at different time intervals. (DOC)

## References

[1] Wassmann R, Jagadish SVK, Heuer S, Ismail A, Redona E, Serraj R, et al Climate change affecting rice production: The physiological and agronomic basis for possible adaptation strategies In Sparks Donald L. (Ed.), Adv. in Agron. 2009 pp. 59–122. 10.1016/S0065-2113(08)00802-X

[2] Agarwal S, Grover A. Isolation and transcription profiling of low-O_2_ stress-associated cDNA clones from the flooding-stress-tolerant FR13A rice genotype. Ann Bot. 2005;96: 831–844. 10.1093/aob/mci233 16115835 PMC4247047

[3] Magneschi L, Perata P. Rice germination and seedling growth in the absence of oxygen. Ann Bot. 2008;103: 181–196. 10.1093/aob/mcn121 18660495 PMC2707302

[4] Vartapetian BB, Jackson MB. Plant adaptations to anaerobic stress. Ann Bot. 1997;79: 3–20.

[5] Liu F, VanToai T, Moy LP, Bock G, Linford LD, Quackenbush J. Global transcription profiling reveals comprehensive insights into hypoxic response in Arabidopsis. Plant Physiol. 2005;137: 1115–1129. 10.1104/pp.104.055475 15734912 PMC1065411

[6] Lasanthi-Kudahettige R, Magneschi L, Loreti E, Gonzali S, Licausi F, Novi G, et al Transcript profiling of the anoxic rice coleoptile. Plant Physiol. 2007;144: 218–231. 10.1104/pp.106.093997 17369434 PMC1913783

[7] Sadiq I, Fanucchi F, Paparelli E, Alpi E, Bachi A, Alpi A, et al Proteomic identification of differentially expressed proteins in the anoxic rice coleoptile. J Plant Physiol. 2011;168: 2234–2243. 10.1016/j.jplph.2011.07.009 21920630

[8] Fujimoto SY, Ohta M, Usui A, Shinshi H, Ohme-Takagi M. Arabidopsis ethylene-responsive element binding factors act as transcriptional activators or repressors of GCC box-mediated gene expression. Plant Cell. 2000;12: 393–404. 10715325 10.1105/tpc.12.3.393 PMC139839

[9] Ohme-Takagi M, Shinshi H. Ethylene-inducible DNA binding proteins that interact with an ethylene-responsive element. Plant Cell.1995; 7:173–182. 10.1105/tpc.7.2.173 7756828 PMC160773

[10] Manners JM, Penninckx IAMA, Vermaere K, Kazan K, Brown RL, Morgan A, et al The promoter of the plant defensin gene PDF1.2 from Arabidopsis is systemically activated by fungal pathogens and responds to methyl jasmonate but not to salicylic acid. Plant Mol Biol. 1998;38: 1071–1080. 10.1023/A:1006070413843 9869413

[11] Saleh A, Pagès M. Plant AP2/ERF transcription factors. Genetika. 2003;35: 37–50. 10.2298/GENSR0301037S

[12] Jung K-H, Seo Y-S, Walia H, Cao P, Fukao T, Canlas PE, et al The submergence tolerance regulator Sub1A mediates stress-responsive expression of AP2/ERF transcription factors. Plant Physiol. 2010;152: 1674–1692. 10.1104/pp.109.152157 20107022 PMC2832257

[13] Sharoni AM, Nuruzzaman M, Satoh K, Shimizu T, Kondoh H, Sasaya T, et al Gene structures, classification and expression models of the AP2/EREBP transcription factor family in rice. Plant Cell Physiol. 2011;52: 344–360. 10.1093/pcp/pcq196 21169347

[14] Kumar A, Smita S, Sahu N, Sharma V, Shankaracharya S, Vidyarthi A, et al In silico analysis of motifs in promoters of differentially expressed genes in rice (Oryza sativa L.) under anoxia. Int J Bioinform Res Appl. 2009;5: 525–547. 10.1504/IJBRA.2009.028681 19778868

[15] Prajapati GK, Pandey DM. Molecular beacon probe based promoter motifs validation in anoxia responsive differentially expressed genes and their in silico interaction studies with AP2/EREBP TF in rice (*Oryza Sativa* L.). Int J Pharm Pharm Sci. 2015;7: 123–130.

[16] Tyagi S, Kramer FR. Molecular beacons: Probes that fluoresce upon hybridization. Nat Biotechnol. 1996;14: 303–308. 10.1038/nbt0396-303 9630890

[17] Andersen CB, Holst-Jensen A, Berdal KG, Thorstensen T, Tengs T. Equal performance of TaqMan, MGB, molecular beacon, and SYBR green-based detection assays in detection and quantification of roundup ready soybean. J Agric Food Chem. 2006;54: 9658–9663. 10.1021/jf061987c 17177484

[18] Carneiro GA, Matić S, Ortu G, Garibaldi A, Spadaro D, Gullino ML. Development and validation of a TaqMan real-time PCR assay for the specific detection and quantification of *Fusarium fujikuroi* in rice plants and seeds. Phytopathology. 2017;107: 885–892. 10.1094/PHYTO-10-16-0371-R 28398878

[19] Lata P, Ram S, Agrawal M, Shanker R. Real time PCR for the rapid detection of vanA gene in surface waters and aquatic macrophyte by molecular beacon probe. Environ Sci Technol. 2009;43: 3343–3348. 10.1021/es803635y 19534156

[20] Täpp I, Malmberg L, Rennel E, Wik M, Syvänen A-C. Homogeneous scoring of single-nucleotide polymorphisms: Comparison of the 5′-nuclease TaqMan assay and molecular beacon probes. Biotechniques. Future Science; 2000;28: 732–738. 10.2144/00284rr02 10769752

[21] Parida MM. Rapid and real-time detection technologies for emerging viruses of biomedical importance. J Biosci. 2008;33: 617–628. 10.1007/s12038-008-0079-7 19208986 PMC7090734

[22] Liu X, Wang X, Jiang H. A steered molecular dynamics method with direction optimization and its applications on ligand molecule dissociation. J Biochem Biophys Methods. 2008;70: 857–864. 10.1016/j.jbbm.2007.10.006 18031823

[23] Brand LH, Fischer NM, Harter K, Kohlbacher O, Wanke D. Elucidating the evolutionary conserved DNA-binding specificities of WRKY transcription factors by molecular dynamics and in vitro binding assays. Nucleic Acids Res. 2013;41: 9764–9778. 10.1093/nar/gkt732 23975197 PMC3834811

[24] Hamzeh-Mivehroud M, Moghaddas-Sani H, Rahbar-Shahrouziasl M, Dastmalchi S. Identifying key interactions stabilizing DOF zinc finger–DNA complexes using *in silico* approaches. J Theor Biol. 2015;382: 150–159. 10.1016/j.jtbi.2015.06.013 26092376

[25] Pandey B, Sharma P, Tyagi C, Goyal S, Grover A, Sharma I. Structural modeling and molecular simulation analysis of HvAP2/EREBP from barley. J Biomol Struct Dyn. Taylor & Francis; 2016;34: 1159–1175. 10.1080/07391102.2015.1073630 26198402

[26] Prajapati GK, Kashyap N, Kumar A, Pandey DM. Identification of GCC box in the promoter region of ubiquinol cytochrome C chaperone gene using molecular beacon probe and its in silico protein-DNA interaction study in Rice (*Oryza sativa* L.). Int J Comput Bioinforma Silico Model. 2013;2: 213–222.

[27] Schwede T, Kopp J, Guex N, Peitsch MC. SWISS-MODEL: An automated protein homology-modeling server. Nucleic Acids Res. 2003;31: 3381–3385. Available: https://www.ncbi.nlm.nih.gov/pubmed/12824332 12824332 10.1093/nar/gkg520 PMC168927

[28] Laskowski R, MacArthur MW, Thornton J. PROCHECK: Validation of protein structure coordinates, In international tables of crystallography, volume F Rossman M.G., Arnold E. (Eds.), Crystallography of Biological Macromolecules. Kluwer Academic Publishers, Dordrecht, The Netherlands 2001 pp. 722–725.

[29] van Dijk M, Bonvin AMJJ. 3D-DART: a DNA structure modelling server. Nucleic Acids Res. 2009;37: W235–W239. 10.1093/nar/gkp287 19417072 PMC2703913

[30] de Vries SJ, van Dijk M, Bonvin AMJJ. The HADDOCK web server for data-driven biomolecular docking. Nat Protoc. 2010;5: 883–897. 10.1038/nprot.2010.32 20431534

[31] Pettersen EF, Goddard TD, Huang CC, Couch GS, Greenblatt DM, Meng EC, et al UCSF Chimera—A visualization system for exploratory research and analysis. J Comput Chem. 2004;25: 1605–1612. 10.1002/jcc.20084 15264254

[32] Abraham MJ, Murtola T, Schulz R, Páll S, Smith JC, Hess B, et al GROMACS: High performance molecular simulations through multi-level parallelism from laptops to supercomputers. SoftwareX. 2015;1–2: 19–25. 10.1016/j.softx.2015.06.001

[33] Hess B, Kutzner C, van der Spoel D, Lindahl E. GROMACS 4: Algorithms for highly efficient, load-balanced, and scalable molecular simulation. J Chem Theory Comput. 2008;4: 435–447. 10.1021/ct700301q 26620784

[34] Yang B, Zhu Y, Wang Y, Chen G. Interaction identification of Zif268 and TATA_ZF_ proteins with GC-/AT-rich DNA sequence: A theoretical study. J Comput Chem. 2011;32: 416–428. 10.1002/jcc.21630 20658568

[35] Wu Y, Tepper HL, Voth GA. Flexible simple point-charge water model with improved liquid-state properties. J Chem Phys. 2006;124: 24503. 10.1063/1.2136877 16422607

[36] Dhanjal JK, Grover S, Sharma S, Singh AK, Grover A. Structural insights into mode of actions of novel natural Mycobacterium protein tyrosine phosphatase B inhibitors. BMC Genomics. 2014;15: S3 10.1186/1471-2164-15-S1-S3 PMC4046716 24564493

[37] Goyal M, Grover S, Dhanjal JK, Goyal S, Tyagi C, Chacko S, et al Novel natural structure corrector of ApoE4 for checking Alzheimer’s disease: benefits from high throughput screening and molecular dynamics simulations. Biomed Res Int. 2013;2013: 620793 10.1155/2013/620793 24324968 PMC3845489

[38] Goyal S, Grover S, Dhanjal JK, Goyal M, Tyagi C, Chacko S, et al Mechanistic insights into mode of actions of novel oligopeptidase B inhibitors for combating leishmaniasis. J Mol Model. 2014;20: 2099 10.1007/s00894-014-2099-6 24567150

[39] Luscombe NM, Laskowski RA, Thornton JM. NUCPLOT: a program to generate schematic diagrams of protein-nucleic acid interactions. Nucleic Acids Res. 1997;25: 4940–4945. 9396800 10.1093/nar/25.24.4940 PMC147160

[40] Hou T, Wang J, Li Y, Wang W. Assessing the performance of the MM/PBSA and MM/GBSA methods. 1. The accuracy of binding free energy calculations based on molecular dynamics simulations. J Chem Inf Model. 2011;51: 69–82. 10.1021/ci100275a 21117705 PMC3029230

[41] Maisuradze GG, Liwo A, Scheraga HA. Principal component analysis for protein folding dynamics. J Mol Biol. 2009;385: 312–329. 10.1016/j.jmb.2008.10.018 18952103 PMC2652707

[42] Sang P, Du X, Yang L-Q, Meng Z-H, Liu S-Q. Molecular motions and free-energy landscape of serine proteinase K in relation to its cold-adaptation: a comparative molecular dynamics simulation study and the underlying mechanisms. RSC Adv. 2017;7: 28580–28590. 10.1039/C6RA23230B

[43] Juven-Gershon T, Kadonaga JT. Regulation of gene expression via the core promoter and the basal transcriptional machinery. Dev Biol. 2010;339: 225–229. 10.1016/j.ydbio.2009.08.009 19682982 PMC2830304

[44] Barsalobres-Cavallari CF, Severino FE, Maluf MP, Maia IG. Identification of suitable internal control genes for expression studies in Coffea arabica under different experimental conditions. BMC Mol Biol. 2009;10: 1 10.1186/1471-2199-10-1 19126214 PMC2629470

[45] Perrody E, Cirinesi A-M, Desplats C, Keppel F, Schwager F, Tranier S, et al A bacteriophage-encoded J-domain protein interacts with the DnaK/Hsp70 chaperone and stabilizes the heat-shock factor σ32 of Escherichia coli. PLOS Genet. 2012;8: e1003037 10.1371/journal.pgen.1003037 23133404 PMC3486835

[46] Zhou W, Zhou T, Li M-X, Zhao C-L, Jia N, Wang X-X, et al The Arabidopsis J-protein AtDjB1 facilitates thermotolerance by protecting cells against heat-induced oxidative damage. New Phytol. 2012;194: 364–378. 10.1111/j.1469-8137.2012.04070.x 22356282

[47] Wang W, Vinocur B, Shoseyov O, Altman A. Role of plant heat-shock proteins and molecular chaperones in the abiotic stress response. Trends Plant Sci. 2004;9: 244–252. 10.1016/j.tplants.2004.03.006 15130550

[48] Wiese A, Elzinga N, Wobbes B, Smeekens S. A conserved upstream open reading frame mediates sucrose-induced repression of translation. Plant Cell. 2004;16:1717–1729. 10.1105/tpc.019349 15208401 PMC514156

[49] Hinnebusch AG. Translational regulation of yeast GCN4: A window on factors that control initiator-trna binding to the ribosome. J Biol Chem. 1997;272: 21661–21664. 10.1074/jbc.272.35.21661 9268289

[50] Hayden CA, Jorgensen RA. Identification of novel conserved peptide uORF homology groups in Arabidopsis and rice reveals ancient eukaryotic origin of select groups and preferential association with transcription factor-encoding genes. BMC Biol. 2007;5: 32 10.1186/1741-7007-5-32 17663791 PMC2075485

[51] Hao D, Ohme-Takagi M, Sarai A. Unique mode of GCC box recognition by the DNA-binding domain of Ethylene-responsive Element-binding Factor (ERF Domain) in plant. J Biol Chem. 1998;273: 26857–26861. 10.1074/jbc.273.41.26857 9756931

[52] Chakravarthy S, Tuori RP, D’Ascenzo MD, Fobert PR, Despres C, Martin GB. The tomato transcription factor Pti4 regulates defense-related gene expression via GCC box and non-GCC box cis elements. Plant Cell. 2003;15: 3033–3050. 10.1105/tpc.017574 14630974 PMC282854

[53] Buttner M, Singh KB. Arabidopsis thaliana ethylene-responsive element binding protein (AtEBP), an ethylene-inducible, GCC box DNA-binding protein interacts with an ocs element binding protein. Proc Natl Acad Sci USA. 1997;94(11):5961–6. 9159183 10.1073/pnas.94.11.5961 PMC20889

[54] andey B, Grover A, Sharma P. Molecular dynamics simulations revealed structural differences among WRKY domain-DNA interaction in barley (Hordeum vulgare). BMC Genomics. 2018;19: 132 10.1186/s12864-018-4506-3 29433424 PMC5810047

[55] Harteis S, Schneider S. Making the bend: DNA tertiary structure and protein-DNA interactions. Int J Mol Sci. 2014;15: 12335–12363. 10.3390/ijms150712335 25026169 PMC4139847

[56] Bavi R, Kumar R, Rampogu S, Son M, Park C, Baek A, et al Molecular interactions of UvrB protein and DNA from *Helicobacter pylori*: Insight into a molecular modeling approach. Comput Biol Med. 2016;75: 181–189. 10.1016/j.compbiomed.2016.06.005 27315565

[57] Tan C, Takada S. Dynamic and structural modeling of the specificity in protein–DNA interactions guided by binding assay and structure data. J Chem Theory Comput. 2018;14: 3877–3889. 10.1021/acs.jctc.8b00299 29806939

[58] Ahmad M, Helms V, Kalinina O V, Lengauer T. Relative principal components analysis: Application to analyzing biomolecular conformational changes. bioRxiv. 2018; 409474 10.1101/409474 PMC6728065 30763093

