# Supplementary figures and images for: Identification of GCC-box and TCC-box motifs in the promoters of differentially expressed genes in rice (Oryza sativa L.): Experimental and computational approaches
Source: PLoS One. 2019 Apr 26;14(4):e0214964. doi: 10.1371/journal.pone.0214964 (PMC6485614; doi:10.1371/journal.pone.0214964)

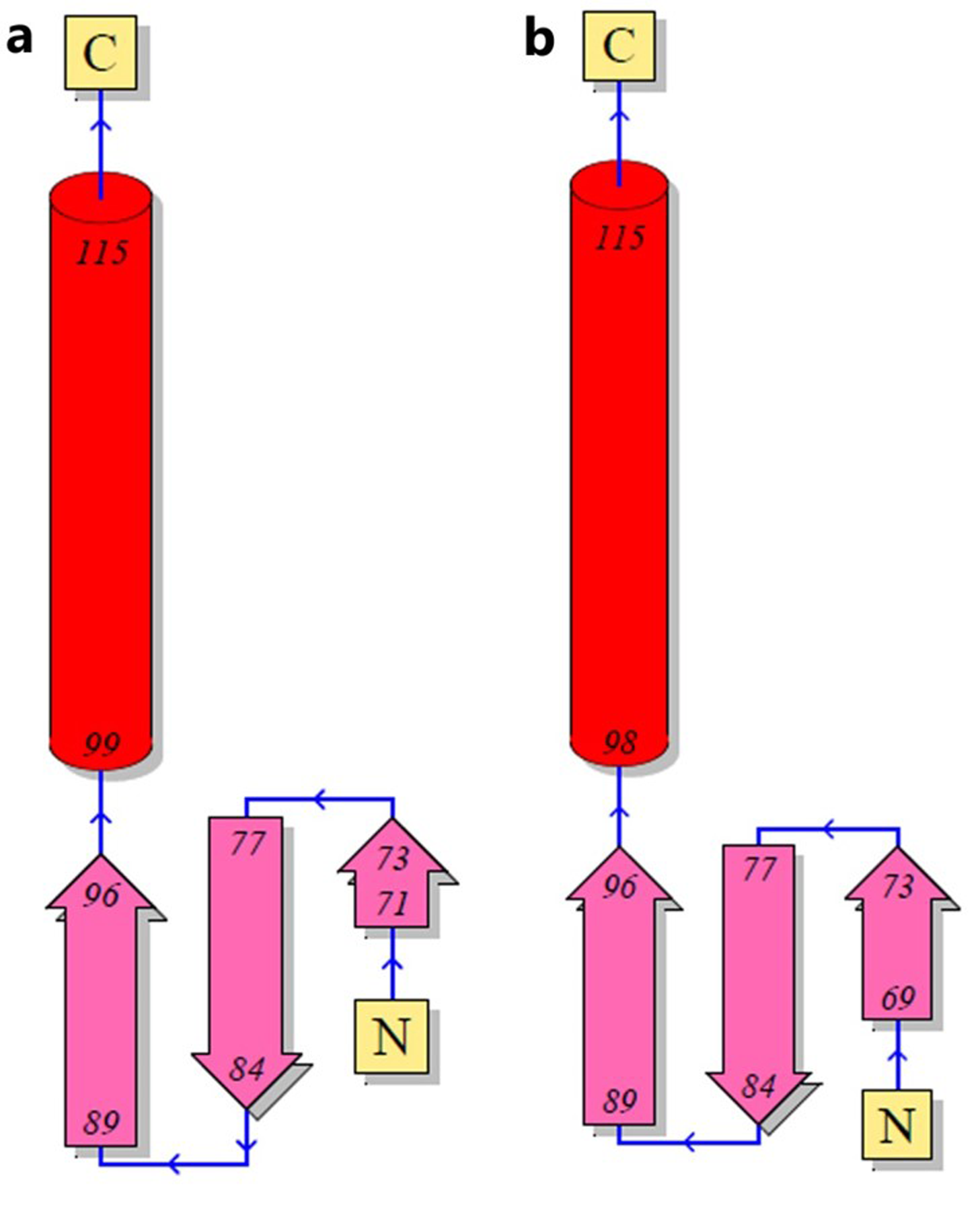

Supplement: S1 Fig — (TIF) [file pone.0214964.s001.tif]

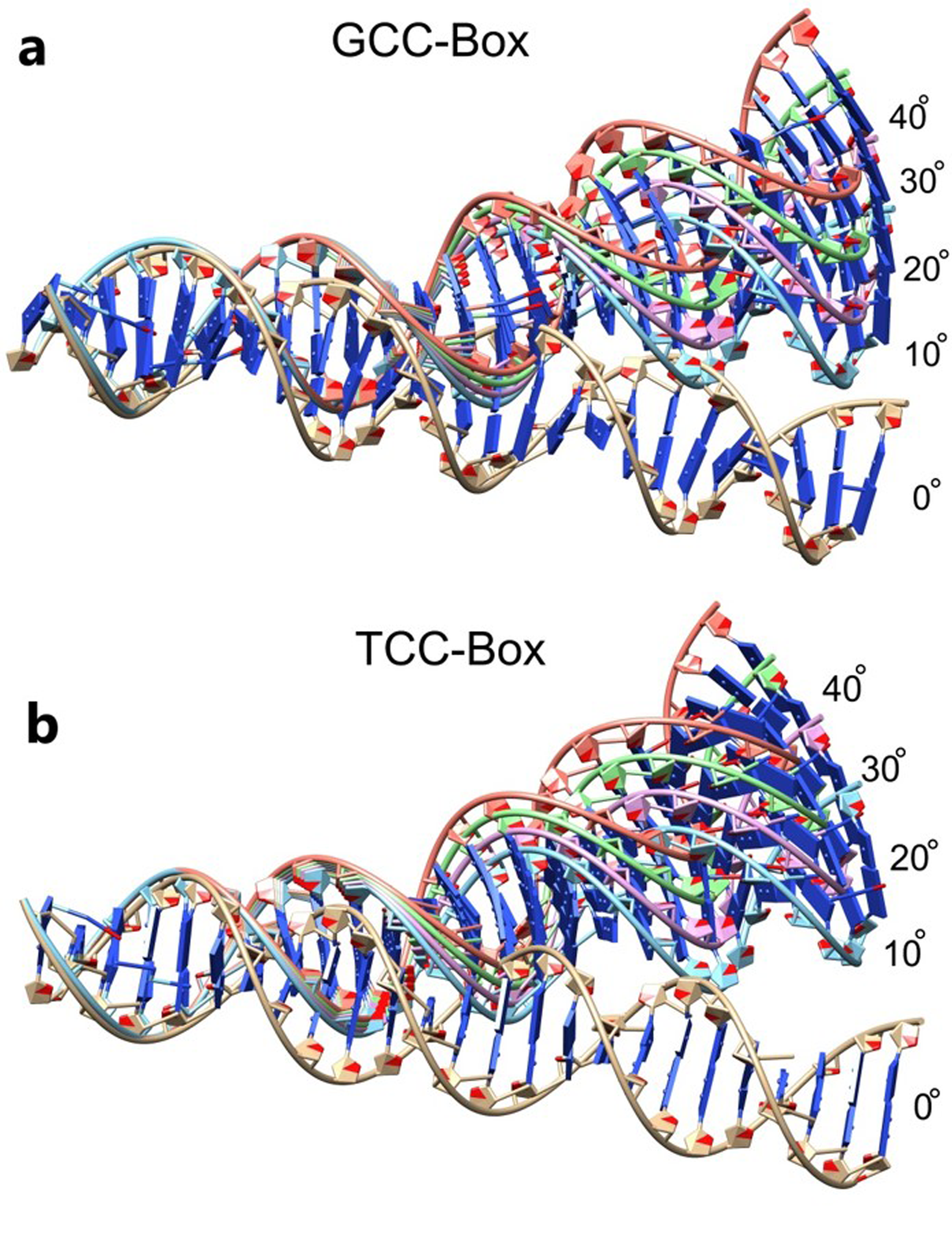

Supplement: S2 Fig — DNA motif bend angle of 0°, 10°, 20°, 30°, and 40° for an (a) GCC-box; and (b) TCC-box. (TIF) [file pone.0214964.s002.tif]

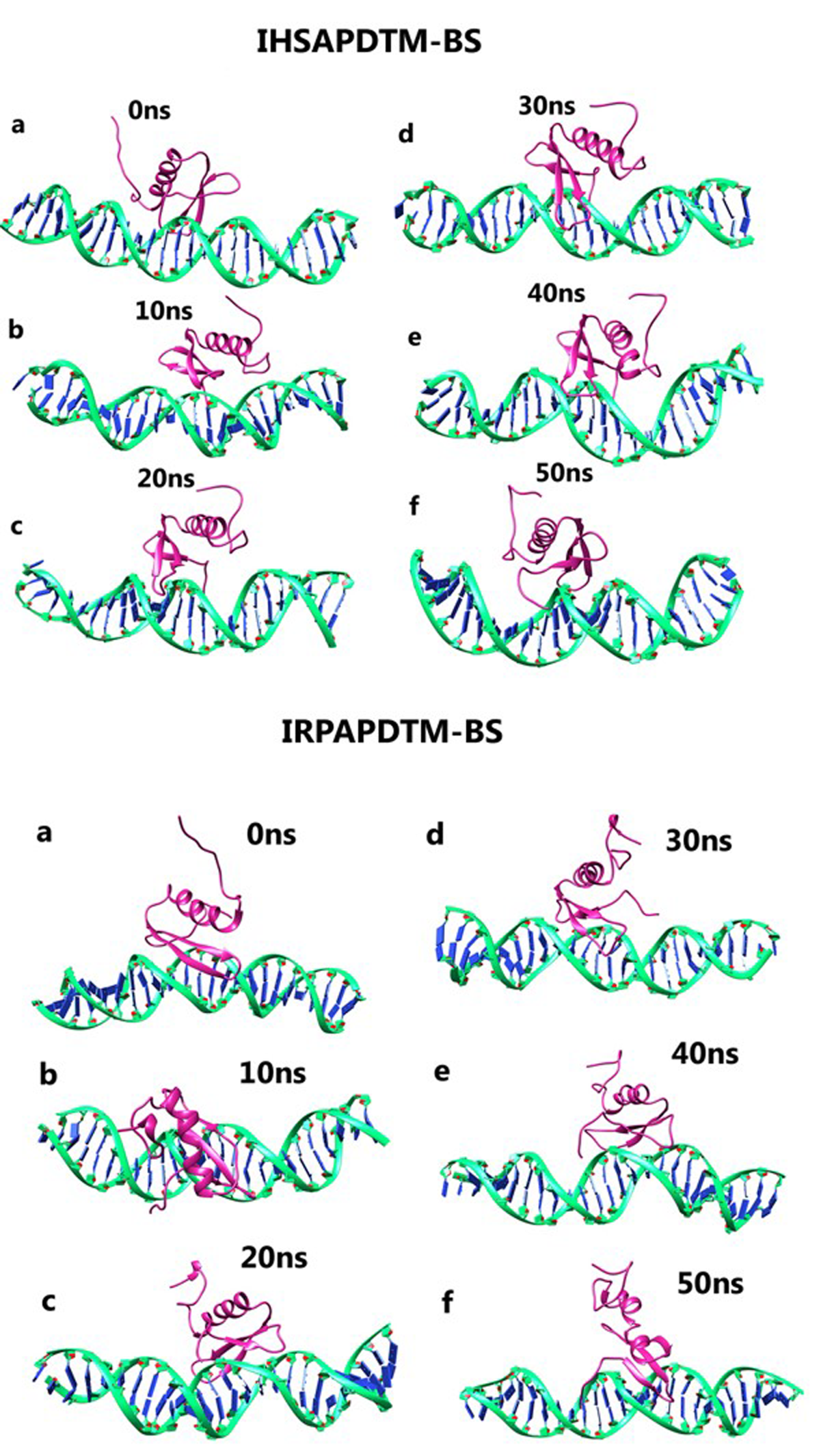

Supplement: S3 Fig — Extracted snapshots of (a) IHSAPDTM-BS and (b) IRPAPDTM-BS complexes at regular intervals during the 50 ns simulation time period. (TIF) [file pone.0214964.s003.tif]

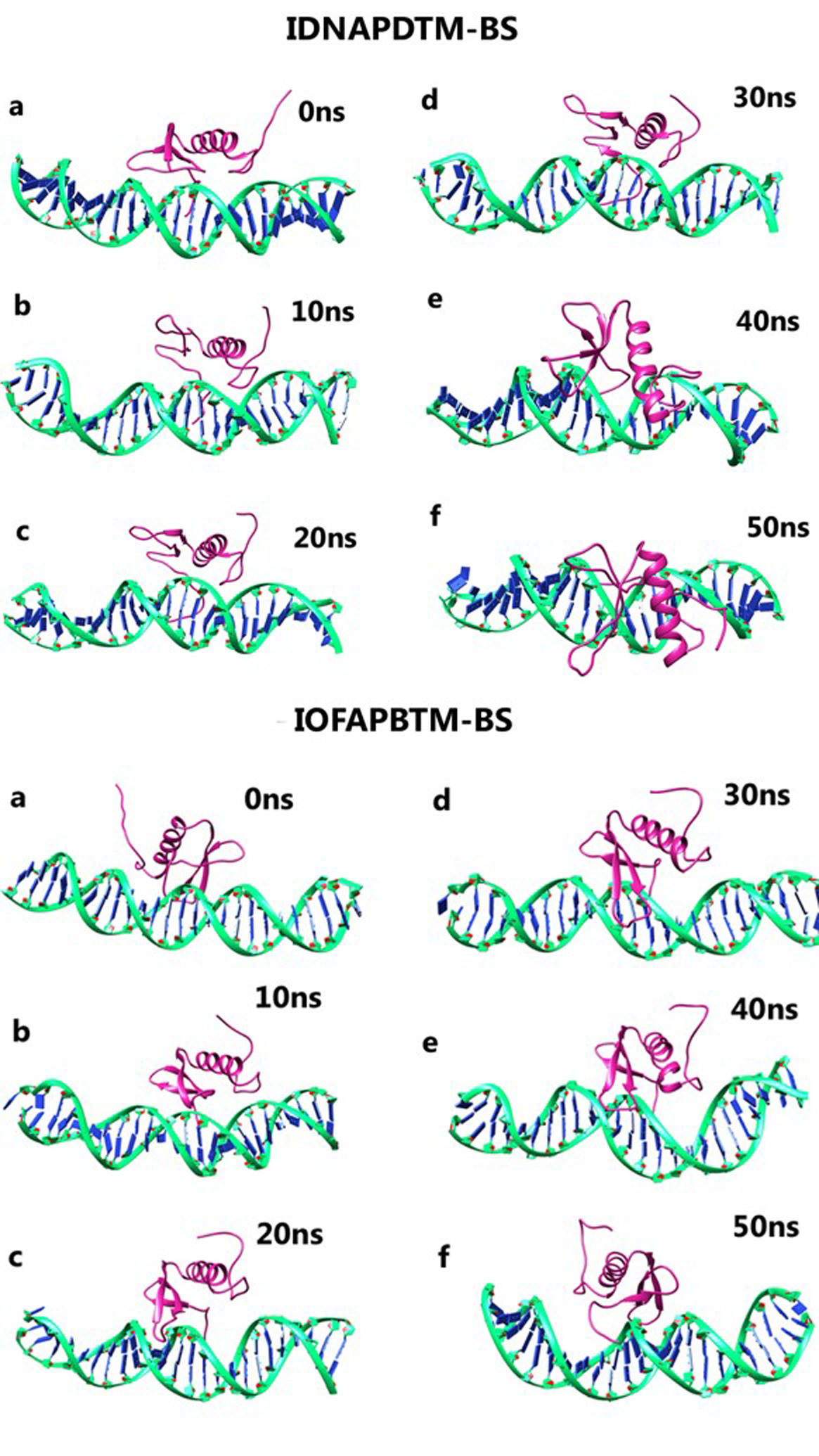

Supplement: S4 Fig — Extracted snapshots of (a) IDNAPDTM-BS and (b) IOFAPBTM-BS complexes at regular intervals during the 50 ns simulation time period. (TIF) [file pone.0214964.s004.tif]

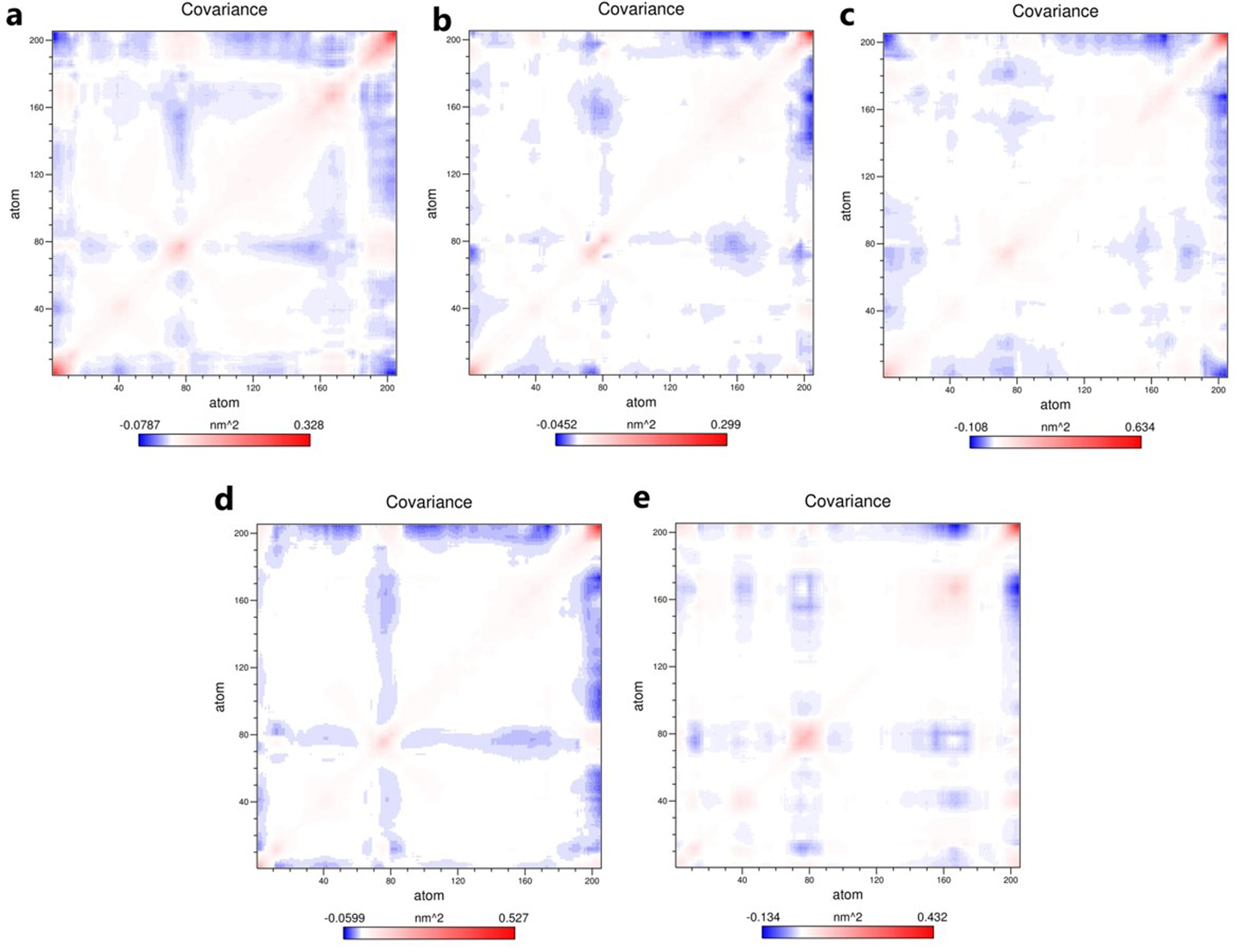

Supplement: S5 Fig — Covariance analysis of the (a) AP2/EREBP TF; (b) IHSAPDTM-BS; (c) IRPAPDTM-BS; (d) IOFAPBTM-BS; and (e) IDNAPDTM-BS. (TIF) [file pone.0214964.s005.tif]
