# Supplementary material for: Identification of GCC-box and TCC-box motifs in the promoters of differentially expressed genes in rice (Oryza sativa L.): Experimental and computational approaches
Source: PLoS One. 2019 Apr 26;14(4):e0214964. doi: 10.1371/journal.pone.0214964 (PMC6485614; doi:10.1371/journal.pone.0214964)
Supplement: S1 Table — (DOCX) [file pone.0214964.s006.docx]

**S1 Table:** RMSD of protein-DNA complexes at different time intervals.

| **Protein-DNA** | **RMSD (nm)** | | |  |  |
| --- | --- | --- | --- | --- | --- |
|  | **10ns** | **20ns** | **30ns** | **40ns** | **50ns** |
| **IHSAPDTM-BS** | 0.56 | 0.60 | 0.60 | 0.47 | 0.49 |
| **IRPAPDTM-BS** | 0.38 | 0.37 | 0.42 | 0.42 | 0.36 |
| **IDNAPDTM-BS** | 0.37 | 0.40 | 0.37 | 0.37 | 0.40 |
| **IOFAPBTM-BS** | 0.39 | 0.40 | 0.47 | 0.51 | 0.43 |
