# Supplementary material for: Identification of GCC-box and TCC-box motifs in the promoters of differentially expressed genes in rice (Oryza sativa L.): Experimental and computational approaches
Source: PLoS One. 2019 Apr 26;14(4):e0214964. doi: 10.1371/journal.pone.0214964 (PMC6485614; doi:10.1371/journal.pone.0214964)
Supplement: S2 Table — (DOC) [file pone.0214964.s007.doc]

**S2 Table:** List of residues involved in the formation of hydrogen bonds in AP2/EREBP TF-DNA complexes at different time intervals.

| **Protein-DNA** | **0ns** | **10ns** | **20ns** | **30ns** | **40ns** | **50ns** |
| --- | --- | --- | --- | --- | --- | --- |
| **IHSAPDTM-BS** | Arg68, Arg73, Lys77, Lys87, Thr95 | Arg68, Arg73, Lys77, Arg83, Lys87, Trp92 | Arg68, Arg73, Arg83, Arg90, Trp92 | Arg72, Arg73, Arg83, Lys87, Thr95 | Arg68, Arg90, Trp92, Thr95 | Arg68, Arg73, Lys87, Arg90, Thr95 |
| **IRPAPDTM-BS** | Arg64, Arg73, Lys77, Arg83 | Arg63, Ag64, Arg73, Arg83, Arg90, Thr95 | Arg63, Ag68, Arg71, Arg83, Arg90, Thr95 | Arg62, Arg64, Gly69, Arg72, Arg73, Arg83, Lys87, Arg90, Trp92, Thr95 | Arg63, Arg64, Arg72, Arg73, Trp75, Lys77 Arg83, Thr95 | Arg71, Arg72, Arg73, Trp75, Arg83, Lys87, Thr95 |
| **IDNAPDTM-BS** | Glu62, Arg63, Arg64, Arg68, Arg71, Arg73, Thr106, Lys119 | Arg63, Arg64, Arg68, Arg71, Arg90, Thr106 | Arg63, Arg64, Arg68, Arg71, Arg83, Thr106, Arg114,  Lys119 | Arg63, Arg64, Thr65, Arg83, Arg90, Thr106,  Lys119 | Arg63, Thr65, Arg68, Arg71, Thr106, Lys117,  Lys119 | Glu62, Arg63, Arg64, Thr65, Arg68, Arg71, Thr106, Lys117 |
| **IOFAPBTM-BS** | Arg64, Arg68, Gly69, Arg71, Arg72, Arg83, Arg90, Lys117, Lys119 | Met1, Arg12, Arg72, Lys77, Arg83, Arg104, Arg108, Arg111, Tyr141, Arg164 | Arg63, Arg64, Arg68, Gly69, Arg72, Arg73, Arg83, Lys119 | Arg63, Arg64, Gly69, Arg71, Arg72, Arg73, Lys117, Lys119 | Arg64, Arg71, Arg72, Arg73, Arg83, Arg90,Arg114, Lys119 | Glu62, Arg63, Arg64, Arg72, Arg83, Lys87, Arg90 |
